# Intratumor heterogeneity and tissue distribution of KRAS mutation in non-small cell lung cancer: implications for detection of mutated KRAS oncogene in exhaled breath condensate

**DOI:** 10.1007/s00432-018-2779-1

**Published:** 2018-10-27

**Authors:** Jacek Kordiak, Janusz Szemraj, Izabela Grabska-Kobylecka, Piotr Bialasiewicz, Marcin Braun, Radzisław Kordek, Dariusz Nowak

**Affiliations:** 1 Department of Chest Surgery, Oncologic and General Surgery, Medical University of Lodz, University Hospital No. 2, Zeromskiego St. 113, 91-647 Lodz, Poland; 2 Department of Medical Biochemistry, Medical University of Lodz, Mazowiecka St. 6/8, 92-215 Lodz, Poland; 3 Department of Clinical Physiology, Medical University of Lodz, Mazowiecka St. 6/8, 92-215 Lodz, Poland; 4 Department of Sleep Medicine and Metabolic Disorders, Medical University of Lodz, Mazowiecka St. 6/8, 92-215 Lodz, Poland; 5 Department of Pathology, Medical University of Lodz, Pomorska St. 251, 92-215 Lodz, Poland

**Keywords:** KRAS oncogene mutation, Heterogeneity, Non-small cell lung cancer, Genetic biomarkers, Exhaled breath condensate

## Abstract

**Purpose:**

Mutated KRAS oncogene in exhaled breath condensate (EBC) can be a genetic marker of non-small cell lung cancer (NSCLC). However, a possibility of inhomogeneous distribution in cancer tissue and intratumor heterogeneity of KRAS mutation may decrease its significance. We investigated a status of KRAS point mutation and its sequence at codon 12 in 51 NSCLC patients after tumor resection. The comparison of KRAS mutation status between EBC–DNA and cancer tissue was performed in 19 cases.

**Methods:**

Five cancer tissue samples from disparate tumor regions and one from normal lung were harvested at surgery. EBC was collected for DNA analysis the previous day. KRAS point mutations at codon 12 were detected using mutant-enriched PCR technique and pyrosequenced.

**Results:**

Forty-six cancers revealed concordance of KRAS mutation status: 27 contained mutated KRAS and 19 had only wild KRAS. Five NSCLCs revealed inhomogeneous distribution of KRAS mutation. Two different mutations were found in 14 NSCLCs and the most frequent one was G12D and G12V (*n* = 8). No mutated KRAS was found in normal lung. The concordance ratios of KRAS sequence in codon 12 between EBC–DNA and cancer were 18/19 for NSCLC patients and 11/12 for KRAS mutation positive NSCLC.

**Conclusions:**

Intratumor heterogeneity and inhomogeneous distribution of KRAS point mutation in codon 12 in cancer tissue can occur in NSCLCs. There was a high accordance between KRAS mutation status in EBC–DNA and cancer tissue in NSCLC patients what suggests usefulness of monitoring KRAS mutation in EBC–DNA as a biomarker of NSCLC.

## Introduction

Exhaled breath condensate (EBC) composed of droplets of airway epithelial lining fluid (ELF) and condensed water vapor contains variety of biomolecules (e.g. proteolytic enzymes, lipid peroxidation products, reactive oxygen and nitrogen species) with potential for non-invasive monitoring of various pathological processes in the airways (Dent et al. 2013; Liang et al. 2012; Kwiatkowska et al. 2012; Stolarek et al. 2010). Discovery of free DNA in EBC together with application of sensitive molecular techniques gave attempt to use EBC as a diagnostic material for assessment of genetic and epigenetic markers of pulmonary malignancy (Gessner et al. 2004). Mutations in proto-oncogenes and suppressor genes (e.g. p53, KRAS, Rb and myc genes) or abnormal promoter methylation belong to molecular mechanisms of malignant transformation and are detected in numerous cancers including non-small cell lung cancer (NSCLC) (Subramaniam et al. 2013). Malignant cells can release DNA via various mechanisms including apoptosis, necrosis or spontaneous active secretion process (Fleischhacker and Schmidt 2007; Pathak et al. 2006). Patients with lung cancer had increased levels of free DNA in blood and partly, this DNA originated from the malignant cells (Pathak et al. 2006; Stroun et al. 1989). DNA released from NSCLC can reach airway lining fluid through the pulmonary microvasculature or can be released directly from the tumor cells into this fluid. Thus, one may expect that some of proto-oncogenes mutations can be detected in DNA isolated from EBC (EBC–DNA) obtained from NSCLC patients. Studies performed within the past few years have confirmed this assumption and showed presence of various DNA-based biomarkers (Xiao et al. 2014; Yang et al. 2013; Carpagnano et al. 2010) including p53 and KRAS gene mutations in EBC of these patients (Gessner et al. 2004; Kordiak et al. 2012). However, some data undermine the value of EBC as a promising source of genetic NSCLC biomarkers. For instance: (a) sequence analysis of p53 gene mutations revealed different point mutations in EBC and corresponding tumor tissue in NSCLC patients (Gessner et al. 2004), (b) although the ratio of mutated to wild KRAS gene in EBC of NSCLC patients decreased after complete tumor resection, this parameter did not correlate with the corresponding ratios in blood and tumor tissue (Kordiak et al. 2012) and (c) comparison of KRAS mutations in excised NSCLC and serum showed higher ratio of positive results in serum than in tumor and only one of 51 studied patients carried the same mutation in both specimens (Ramirez et al. 2003). These discrepancies can result either from coexistence of other extra-tumor sources of mutated oncogenes or intratumor heterogeneity of these mutations. They can limit the usefulness of EBC as a diagnostic material for sensitive and specific monitoring of selected genetic biomarkers of pulmonary malignancy. Therefore, to solve this issue we decided to conduct a prospective study on plausible intratumor heterogeneity of KRAS oncogene point mutations at codon 12 in five different NSCLC samples from each cancer specimen, collected according to standardized protocol from 51 tumors after their complete surgical resection. Furthermore, to compare KRAS mutations in DNA extracted from pre-surgery EBC, blood samples and cancer tissue in 19 NSCLC patients were collected.

## Materials and methods

### Study population

The study conducted from September 2012 to October 2015 involved 51 patients with diagnosis of NSCLC qualified for tumor resection at the Department of Chest, Oncologic and General Surgery, University Teaching Hospital No. 2 in Lodz. The exclusion criteria included a history of other malignancy, any previous potentially mutagenic treatment (e.g. radiotherapy, chemotherapy) or any active infectious disease. All patients underwent standard diagnostic and staging procedures as previously described (Kordiak et al. 2012). Sixteen patients had a diagnosis of adenocarcinoma (ADC) and 32 of squamous cell carcinoma (SCC), two of large cell neuroendocrine carcinoma and one of adenosquamous cell carcinoma based on histological examination of resected tumor (Table 1). The post-surgery TNM classification of NSCLCs (Detterbeck et al. 2013) was as follows: IA-1, IB-4, IIA-3, IIB-4, IIIA-3 and stage IIIB-1 subject in the subgroup of ADC patients; IA-2, IB-5, IIA-6, IIB-8, IIIA-10 and stage IIIB-1 subject in the SCC subgroup.

**Table 1:** Characteristics of studied group of patients with non-small cell lung cancer

Demographic/clinical variable	Patients with non-small cell lung cancer		
	Squamous cell carcinoma	Adenocarcinoma	Whole group
Number	32	16	51^b^
Sex F/M	15/17	6/10	24/27
Age (years)	67 ± 8	66 ± 8	66 ± 8
Surgery Pn/Bl/Lo	7/0/25	3/4/9	12/4/35
Cs/Fs/Ns	15/13/4	8/5/3	25/18/8
Pack-years^a^	38 ± 20	40 ± 21	39 ± 20

*Pn* pneumonectomy, *Bl* bilobectomy, *Lo* lobectomy, *Cs* current smokers, *Fs* former smokers, *Ns* never smokers

^a^Cumulative cigarette consumption—calculated for Cs and Fs together

^b^Additionally one adenosquamous cell carcinoma and two large cell neuroendocrine carcinomas, post-surgery classification IIB, IB and IIIA, respectively. Data presented as a mean and standard deviation, where applicable

### Study protocol

**Fig. 1:**
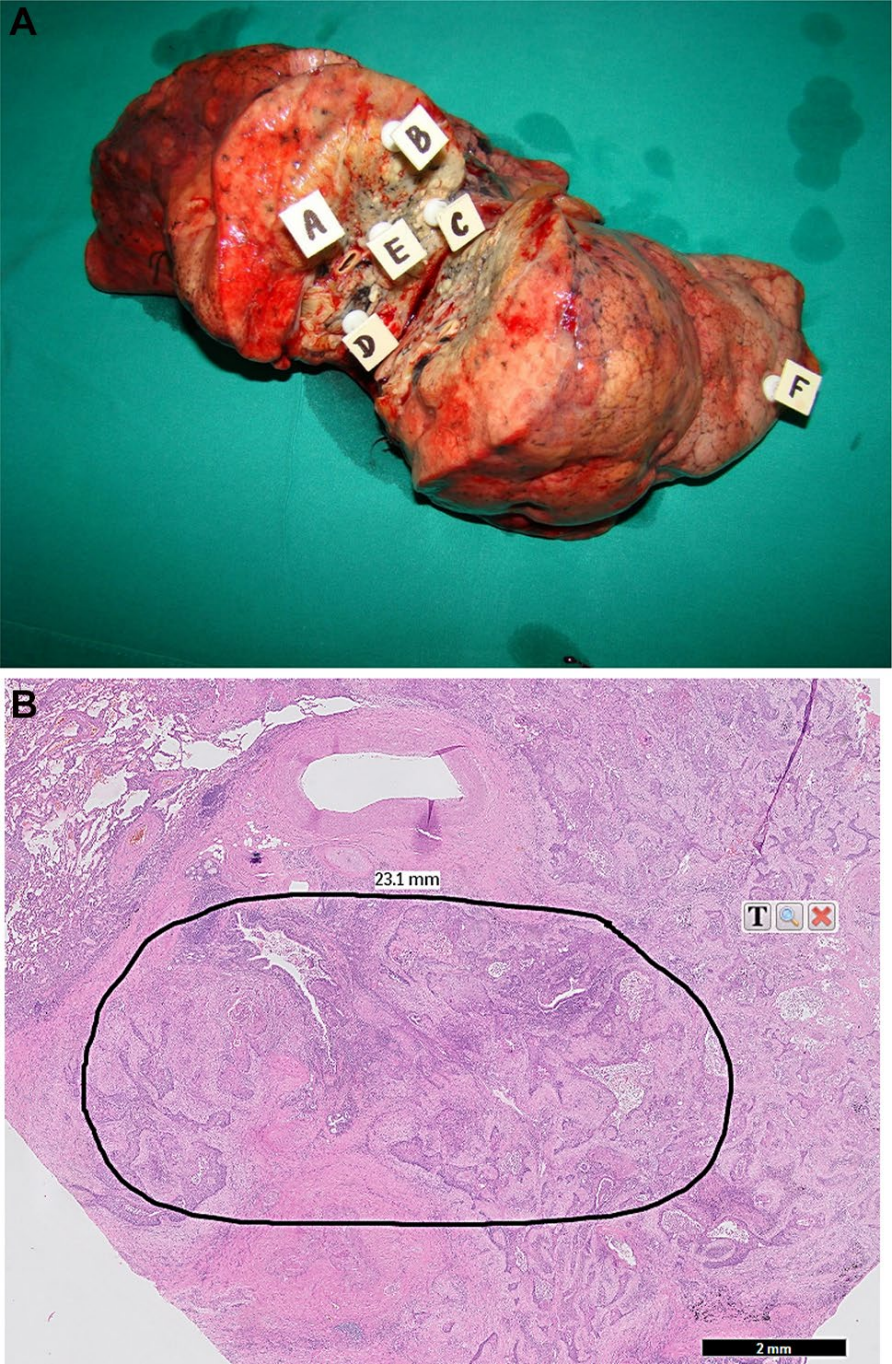
**a** Cross section of resected right lung with squamous cell carcinoma from a 74-year-old male patient. White squares A, B, C, D and E indicate loci of cancer tissue harvesting (samples of about 60–100 mg of wet mass). Normal lung parenchyma was collected from the place marked with square F. **b** Hematoxylin–eosin stained slide (fivefold magnification) prepared from a cancer tissue sample collected from locus A. The black perimeter shows the zone containing more than 90% of cancer cells which was excised for DNA isolation and KRAS mutation analysis. Slides prepared from cancer samples collected from remaining four loci revealed very similar histological images

Resected lung parenchyma (i.e. a lobe or a lung) with tumor was cut transsectionally to obtain two cross sections of the lesion. Afterwards, from the one randomly chosen cross section five samples of cancer tissue (about 60–100 mg of wet mass) were harvested from selected regions of the tumor according to the scheme shown on Fig. 1. The distance between sampled areas was between 0.5 and 3.0 cm depending on the size of the lesion. Additionally, one sample of the normal lung parenchyma was collected as distant as possible from the tumor. Each sample was harvested with the separate set of surgical instruments, washed in ice-cold sterile 0.9% NaCl and desiccated with lignin. Afterwards, samples were fixed with formalin and tissue paraffin blocks were prepared. Overall, 306 formalin fixed, paraffin-embedded (FFPE) blocks (five tumor tissue blocks and one normal lung parenchyma block per patient) were used for preparation of hematoxylin-eosin stained slides that were evaluated by an experienced pathologist to select and mark an area of tumor tissue containing at least 90% cancer cells and pulmonary tissue free of any pathology for subsequent isolation of cancer and normal lung tissue core (a diameter of about 1.5 mm) and DNA extraction. Additionally, EBC and blood specimens were collected the day before surgery in 19 randomly selected patients (Table 2). The Ethics Committee of Medical University of Lodz approved the study protocol (RNN/55/12/KB) and all participants provided a written, informed consent.

**Table 2:** Characteristics of patients with non-small cell lung cancer who donated blood and exhaled breath condensate samples for KRAS mutations analyses

Demographic/clinical variable	Patients with non-small cell lung cancer		
	Squamous cell carcinoma	Adenocarcinoma	Whole group
Number	9	7	19^b^
Sex F/M	5/4	2/5	10/9
Age (years)	67 ± 10	70 ± 6	67 ± 9
Surgery Pn/Bl/Lo	1/0/8	2/1/4	5/1/13
Cs/Fs/Ns	4/4/1	3/3/1	9/7/3
Pack-years^a^	31 ± 17	38 ± 20	35 ± 17

*Pn* pneumonectomy, *Bl* bilobectomy, *Lo* lobectomy, *Cs* current smokers, *Fs* former smokers, *Ns* never smokers

^a^Cumulative cigarette consumption—calculated for Cs and Fs together

^b^Additionally one adenosquamous cell carcinoma and two large cell neuroendocrine carcinomas. Data presented as a mean and standard deviation, where applicable

### Collection of exhaled breath condensate (EBC) and blood samples

EBC samples (3–4 ml) were collected during 25 min of spontaneous tidal volume breathing using EcoScreen-1 (Erich Jaeger GmbH, Hoechberg, Germany) with saliva trap. The collecting temperature of the condenser was − 20 °C. Subjects wore a nose clamp and rinsed their mouth with distilled water (100 ml) just before and after 7 and 14 min of collection procedure to prevent EBC contamination with saliva and droplets of secretions of the nasal mucosa. Along with the EBC, a venous blood specimen (3 ml, EDTA-tubes) was taken. All collections were done the day before surgery between 8:30 and 10:30 a.m. and EBC specimens were immediately transferred to Eppendorf tubes and frozen.

### DNA extraction and detection of KRAS oncogene point mutations at codon 12

Genomic DNA was extracted from FFPE cancer and normal lung tissue core as well as from EBC (previously lyophilized 3 ml specimen) and blood samples (0.3 ml) with the ReliaPrep™ FFPE gDNA Miniprep System (Promega Corp. Madison, WI, USA). Isolated DNA was dissolved in low TE buffer (5 mM Tris, 0.5 mM EDTA, pH 8.0) to final concentration of 25 ng/µl and stored at − 20 °C until the assay. A mutant-enriched PCR (MEPCR) technique of detection of point mutations at codon 12 of KRAS oncogene was used (Nollau et al. 1996). Briefly, 2 µl of isolated DNA solution (50 ng) was added to 23 µl of reaction solution containing PCR GoTaq polymerase buffer, 1.5 mM MgCl_2_, 0.08 mM dNTPs, 2 U Pfu DNA polymerase (Promega Corp Madison, WI, USA) and 0.1 µM of the primers KRAS 3F (5′-ACTGAATATAAACTTGTGGTAGTTGGACCT-3′) and KRAS 10B (5′-ACTCATGAAAATGGTCAGAGAAACCTTTAT-3′). PCR was carried out using Biometra thermal cycler with an initial pre-PCR heat step at 95 °C for 5 min for polymerase activation, followed by 20 cycles of 95 °C for 30 s, annealing at 60 °C for 30 s and extension at 72 °C for 30 s. This was followed by a final step at 72 °C for 10 min. Subsequently, 8 µl of the PCR product was transferred to a new test tube containing NEBuffer 2, bovine serum albumin (200 µg/ml), 2 U BstNI restriction enzyme (New England BioLabs, Beverly, MA, USA) and incubated at 60 °C overnight. Then 4 µl of the restriction digest was amplified in a second PCR run in a final volume of 25 µl with PCR Gold Taq buffer, 1.5 mM MgCl_2_, 200 µM dNTPs, 2 U Pfu DNA polymerase (Promega Corp Madison, WI, USA) and 0.5 µM of the primers KRAS 3F and KRAS 14B (5′-TCAAAGAATGGTCCTGGACC-3′). PCR was cycled at 95 °C for 5 min, followed by 40 cycles of 95 °C for 30 s, annealing at 60 °C for 30 s, extension at 72 °C for 30 s and final extension at 72 °C for 10 min. Amplified product (15 µl) was digested a second time with 5 U BstNI at 60 °C overnight. The final digestion product was electrophoresed on a 6% non-denaturing polyacrylamide gel and 3% agarose gel, then stained with ethidium bromide and visualized by UV transillumination with the Image Master VDS gel documentation system (Pharmacia Biotech), sensitivity of 76 pg DNA per band (ImageMaster VDS Application Note #1  1997). Mutant KRAS DNA was evident on the gel as a single band of 142 bp length; failure of digestion was evident as a band at 157 bp and cleaved, non-mutated (wild type) KRAS DNA was evident as a band at 113 bp (Kordiak et al. 2012). When the band at 157 bp was visible, the result was rejected and all MEPCR steps were repeated. Three separate MEPCR runs were done with DNA samples from each FFPE tissue core along with control systems and in all cases they gave the same results. The control systems included: (1) DNA-free PCR mix -negative control, (2) wild KRAS DNA fragment (113 bp) and (3) mutant KRAS DNA fragment (142 bp) at codon 12 (valine mutation, GTT) prepared as previously described (Kordiak et al. 2012)–positive control.

### Sequence analysis of KRAS oncogene mutations

KRAS exon 2 in DNA of all FFPE cancer and lung tissue cores was amplified with the use of the following primers: KRAS 17F (5′-TGGTGGAGTATTTGATAGTGTA-3′), KRAS 18B (5′-CATGAAAATGGTCAGAGAA-3′). The PCR products were purified to eliminate unincorporated primers and dNTPs and sequenced (pyrosequencing, GS FLX Titanium 454, Roche Diagnostics) in Sequencing Service of Institute of Biochemistry and Biophysics in Warsaw.

### Statistical analysis

Chi square test with Yates’ correction was applied for testing the differences in distribution of KRAS point mutations at codon 12 in tumor DNA of ADCs and SCCs as well as for comparison of frequencies of mutated sequences in all (*n* = 149) analyzed NSCLC samples harboring KRAS mutation. Spearman’s *r* was used for analysis of correlations between the number of different mutated sequences present in the cancer and selected clinical and demographic variables. To test differences in frequencies of Bayesian variables *Z* statistics was used. A *p* value < 0.05 was considered significant.

## Results

### KRAS mutation status in NSCLC samples

Twenty-seven of 51 NSCLCs revealed mutated KRAS gene (point mutations at codon 12) in all five samples. In five cases we observed inhomogeneous distribution of KRAS point mutation: up to four samples contained mutated KRAS oncogene while in the remaining ones only wild (unmutated) KRAS gene was detected (Table 3). The remaining 19 NSCLCs had no detectable KRAS mutation in all samples. None of 51 corresponding specimens of normal lung parenchyma contained detectable KRAS mutation at codon 12. The frequency of KRAS mutation in ADCs and SCCs was 0.81 and 0.50 (overall 0.63, 95% CI 0.46–0.80), respectively, and did not differ significantly from each other (*p* = 0.076).

**Table 3:** Patients with detectable KRAS oncogene point mutations in codon 12 in at least one of five analyzed cancer tissue samples with occurrence of homogenous or inhomogeneous distribution of KRAS mutation over the cancer tissue

Fraction of KRAS mutation positive cancer samples	Number of NSCLC patients	Histological diagnosis	KRAS mutation distribution over the cancer tissue	
		SSC/ADC/ADSCC/LCNEC	Homogeneous	Inhomogeneous
5/5	27	16/10/0/1	27	0
4/5	2	0/2/0/0	0	2
3/5	1	0/0/0/1	0	1
2/5	1	0/1/0/0	0	1
1/5	1	0/0/1/0	0	1
Overall	32	16/13/1/2	27	5

Thirty-two of 51 NSCLCs revealed mutated KRAS gene (point mutations at codon 12) in at least one of all five samples harvested at surgery. Inhomogeneous distribution of KRAS point mutation was noted when not all of five analyzed samples of cancer tissue had detectable mutation

*NSCLC* non-small cell lung cancer, *SCC* squamous cell carcinoma, *ADC* adenocarcinoma, *ADSCC* adenosquamous cell carcinoma, *LCNEC* large cell neuroendocrine carcinoma

### Variability of mutated sequences of KRAS oncogene point mutations at codon 12 in NSCLCs samples

Between-tumors comparison of mutated sequences revealed important variability (Table 4). Five different point mutations at codon 12 of KRAS oncogene were identified: G12D, G12V, G12C, G12A and G12S. Overall, the mutated KRAS oncogene was found in 149 of 255 samples obtained from 51 NSCLCs. The most frequent point mutation was G12D (90/149) and the most uncommon was G12S (3/149) (*p* < 0.001).

**Table 4:** Intratumor heterogeneity of KRAS oncogene point mutations in codon 12 in the group of 31 NSCLC patients harboring this mutation in at least 2 of 5 separate cancer tissue samples obtained after complete tumor resection

Number of patients	Fraction of KRAS mutation positive cancer tissue samples	Histological diagnosis SCC/ADC/LCNEC	Number of identified KRAS point mutations in KRAS mutation positive cancer tissue samples				
			G12D	G12V	G12C	G12A	G12S
Patients presenting with intratumor heterogeneity of KRAS oncogene point mutations (*n* = 14)							
5	5/5	2/3/0	2	3	0	0	0
3	5/5	3/0/0	3	2	0	0	0
2	5/5	2/0/0	0	3	2	0	0
2	5/5	2/0/0	2	0	3	0	0
1	5/5	1/0/0	2	0	0	0	3
1	5/5	0/1/0	1	0	4	0	0
Patients not presenting with intratumor heterogeneity of KRAS oncogene point mutations (*n* = 17)							
10	5/5	4/5/1	5	0	0	0	0
2	5/5	1/1/0	0	0	0	5	0
1	5/5	1/0/0	0	5	0	0	0
2	4/5	0/2/0	4	0	0	0	0
1	3/5	0/0/1	3	0	0	0	0
1	2/5	0/1/0	2	0	0	0	0

Intratumor heterogeneity of mutated sequence was noted when at least two different mutated sequences were detected in analyzed samples of cancer tissue. One patient with adenosquamous cell carcinoma who had KRAS point mutation G12D in one of five cancer tissue samples is not shown in the table

*NSCLC* non-small cell lung cancer, *SCC* squamous cell carcinoma, *ADC* adenocarcinoma, *LCNEC* large cell neuroendocrine carcinoma

### Between-sample comparison of point mutations sequence at codon 12 in NSCLCs harboring mutated KRAS oncogene

Thirty-one NSCLCs revealed mutated KRAS gene in at least two of five analyzed samples (Tables 3, 4). Sequence analysis of KRAS oncogene point mutations revealed no between-samples differences in 17 NSCLCs harboring this mutation. They had the same point mutation in all specimens positive for KRAS mutation at codon 12 (Table 4). Fourteen NSCLCs revealed intratumor heterogeneity of KRAS point mutation defined as the coexistence of two different mutations in cancer tissue. The following pairs of coexistent point mutations were sequenced: G12D and G12V (*n* = 8); G12D and G12C (*n* = 3); G12V and G12C (*n* = 2); G12D and G12S (*n* = 1).

### Occurrence of KRAS mutated gene in blood and EBC samples in relation to KRAS mutation status in NSCLC tissue samples

Seven patients from the group of 19 NSCLC subjects who donated blood and EBC samples had no detectable KRAS mutation in all analyzed tumor samples. All of them had also KRAS mutation negative EBC samples (data not shown) while two patients revealed positive result in blood (Table 5, patient nos. 4 and 11). In 11 EBC samples obtained from the remaining subgroup of 12 KRAS mutation positive NSCLC patients’ samples the same mutated sequence was observed as in the cancer tissue (Table 5). The only one case of discrepancy (patient no. 3, Table 5) between EBC and cancer tissue resulted from the wild KRAS sequence in EBC sample. However, more discrepancies (*n* = 7) were observed between blood and positive cancer tissue for mutated KRAS sequences. In five cases the wild blood KRAS sequence was observed (patients nos. 1, 2, 7, 9 and 10, Table 5) while in two (patients nos. 6 and 12, Table 5) the blood mutated KRAS sequence differed from those found in cancer tissue. Two additional discrepancies (blood positive for KRAS mutation and cancer samples negative for KRAS mutation) were noted in two cases (patients 4 and 11, Table 5). The concordance ratios of KRAS sequences were 11/12 and 5/12 (*p* = 0.03) between EBC and cancer tissue, and blood and cancer tissue, respectively, in the subgroup of 12 KRAS-mutation positive NSCLC patients. Consequently, the concordance ratio for EBC versus blood was 4/12. Overall, these ratios calculated for the whole group of 19 NSCLC patients (7 KRAS-mutation negative and 12 KRAS-mutation positive) were 18/19 (EBC versus cancer), 10/19 (blood versus cancer) and 9/19 (EBC versus blood), respectively (*p* = 0.001).

**Table 5:** Discrepancies between results of analysis of KRAS oncogene point mutations in codon 12 in exhaled breath condensate (EBC), blood and five separate samples of non-small cell lung cancer obtained from the group of 14 patients with clinical diagnosis of lung cancer after complete tumor resection who harbored this mutation in at least one sample

Patient number and diagnosis	Identified KRAS point mutations			Number of discrepancies		
	EBC	Blood	Cancer tissue samples^a^	EBC versus blood	EBC versus tumor	Blood versus tumor
1. SCC	G12D	(–)	3 G12D, 2 G12V	1	0	1
2. SCC	G12D	(–)	5 G12D	1	0	1
3. SCC	(–)	G12C	3 G12V, 2 G12C	1	1	0
4. SCC	(–)	G12D	5 (–)	1	0	1
5. ADC	G12D	G12D	5 G12D	0	0	0
6. ADC	G12D	G12V	5 G12D	1	0	1
7. ADC	G12D	(–)	3 G12V, 2 G12D	1	0	1
8. ADC	G12D	G12D	5 G12D	0	0	0
9. ADC	G12D	(–)	4 G12D, 1 (–)	1	0	1
10. ADC	G12D	(–)	2 G12D, 3 (–)	1	0	1
11. ADC	(–)	G12D	5 (–)	1	0	1
12. ADSCC	G12D	G12V	1 G12D, 4 (–)	1	0	1
13. LCNEC	G12D	G12D	5 G12D	0	0	0
14. LCNEC	G12D	G12D	3 G12D, 2 (–)	0	0	0
Overall				10	1	9

Five patients who had no detectable KRAS point mutations in any analyzed sample were not shown in the table. The discrepancy between EBC and tumor as well as between blood and tumor was noted when KRAS mutation status (including the wild, not mutated sequence) detected in EBC and blood differed from mutation (or mutations) observed in corresponding set of five cancer tissue samples. The discrepancy between EBC and blood was noted when different mutations or mutation and wild-type sequence were observed

*SCC* squamous cell carcinoma, *ADC* adenocarcinoma, *ADSCC* adenosquamous cell carcinoma, *LCNEC* large-cell neuroendocrine carcinoma, *(–)* KRAS mutation not detected

^a^Numeral before mutation represents number of cancer tissue samples harboring given point mutation

### The value of EBC in diagnostics of KRAS mutation status of NSCLC; a Bayesian analysis

We assessed KRAS mutation status in EBC against its presence in tumor tissue. From the group of 12 patients with KRAS mutation found in tumor, only one had negative EBC (a false negative). There were seven patients negative for the mutation in tumor tissue and all of them had negative EBC (i.e. there were no false positives). Therefore, the calculated sensitivity reached 1.0% (95% CI 1.0–1.0) and specificity 0.86 (95% CI 0.63–1.12). Thus, KRAS positive EBC changed probability of the presence of this mutation in tumor from 0.63 (95% CI 0.36–0.91) to 0.92 (95% CI 0.75–1.0, PPV), *p* = 0.04. Conversely, KRAS negative EBC resulted in NPV of 1.0 (95% CI 1.0–1.0), *p* = 0.003, a change from pre-test probability of 0.37.

### Correlations between KRAS mutations and clinical variables

The number of different KRAS point mutations at codon 12 (according to the sequence analysis) was 1 and 2 in 18 and 14 NSCLCs, respectively, while no difference was found in 19 cases. There was no correlation between the number of different KRAS mutations and post-surgery cancer staging (*R* = 0.06, *p* = 0.65), tumor size according to TNM classification (*R* = 0.1, *p* = 0.49), cumulative cigarette consumption expressed as pack-years (*R* = − 0.02, *p* = 0.90) or patients’ age (*R* = − 0.04, *p* = 0.76). Similar results were observed when these correlations were separately calculated for ADC or SCC subgroups (data not shown). The frequency of two coexistent KRAS point mutations in cancer tissue did not associate with the histological type and was similar for ADC (4/16) and SCC (10/32) (*p* = 0.9).

## Discussion

We detected KRAS mutations at codon 12 in 32/51 (62.7%) NSCLCs. This value is higher than ratio of 0.25 reported in a recent study that involved analysis of 7 different base substitutions at codons 12 and 13 of KRAS in NSCLC from 1765 patients (Maus et al. 2014). In other studies based on analysis of 207, 832 and 218 NSCLCs, 34%, 33% and 23.4% tumors harbored KRAS mutations, respectively (Yamaguchi et al. 2013; Smits et al. 2012; Rulli et al. 2015). In addition, genotyping of 3026 ADCs revealed the frequency of KRAS mutations of 0.34 in tumors resected from cigarette smokers (Dogan et al. 2012). Lower number of analyzed patients in comparison with the aforementioned studies and hence, the broader 95% confidence interval of the mean could be the cause of surprisingly high ratio of KRAS mutations reported in our study. Similarly, recent report showing KRAS mutations in 9 of 20 analyzed ADCs and no mutations in 10 studied SCCs (Alsdorf et al. 2013) is in conformity with this explanation. Moreover, analysis of 77 samples of brain metastases from NSCLCs revealed 38.5% frequency of KRAS mutations (Villalva et al. 2013). On the other hand, the parallel analysis of five excision specimens of tumor with cancer cellularity > 90% per one NSCLC patient may contribute to high frequency of KRAS mutations observed in our study. For instance Sutton et al. (2017) reported 30.7% frequency of KRAS mutation in NSCLC on the basis of genotyping of 179 samples, majority of which were thin caliber needle cores with minimum tumor cellularity set at 20%. It should be pointed out that 51 samples of corresponding uninvolved lung parenchyma revealed no KRAS mutation at codon 12. This essentially excludes the possibility of false positive results of genetic analysis that could have contributed to the high ratio of KRAS mutations in our study. On the other hand, numerous hotspot cancer gene mutations (including KRAS mutation) were recently detected in EBC–DNA obtained from healthy subjects (cigarette smokers and nonsmokers) using the more sensitive amplicon-based next-generation sequencing method (Youssef et al. 2017). Therefore, the different frequencies of KRAS mutations in NSCLC described by various authors may also result from various sensitivities of applied methods of mutation detection. For analysis of KRAS mutation status we employed mutant-enriched PCR technique with subsequent analysis of DNA electropherograms using Image Master VDS that amplifies many times the sensitivity of DNA detection and enabled to detect the ratio of mutated KRAS to wild KRAS in a mixture of PCR products even lower than 1:200 (Kordiak et al. 2012). Moreover, all positive results were confirmed by the direct sequence analysis.

### Distribution of KRAS mutation in the cancer tissue

Twenty-seven of 32 NSCLCs harboring KRAS mutations contained mutated KRAS in all five samples from different regions. Five tumors lacked KRAS mutation in at least one sample being positive in the remaining ones. These suggest that KRAS mutations at codon 12 are widespread over the whole cancer tissue and probably, inhomogeneous distribution of KRAS point mutation in NSCLC is relatively rare (5/32 of studied KRAS mutation positive tumors). These results are in accordance with the previous reports showing homogeneous distribution of KRAS mutation status in retrospectively studied, paraffin-embedded tumor samples from NSCLC patients subjected to surgical treatment (Alsdorf et al. 2013; Li et al. 1994).

### Intratumor heterogeneity of KRAS point mutation at codon 12 according to sequence analysis

Fourteen of 31 tumors harboring KRAS mutation at codon 12 in at least 2 out of 5 analyzed samples revealed intratumor heterogeneity of this mutation defined as the coexistence of two different mutated sequences in cancer tissue. Codon 12 is recognized as the “hotspot mutation region” having inherent instability and predisposition to single nucleotide substitutions (Myers et al. 2012) with the result that majority of KRAS mutations occurs at this locus (Okudela et al. 2010). It cannot be excluded that after one point mutation this region remains instable and prone to the occurrence of successive mutations. The majority of studied patients were current or former cigarette smokers. Therefore, cancer cell clones harboring KRAS mutation could be chronically exposed to cigarette smoke-derived mutagens with subsequent induction of KRAS mutation/mutations at already mutated sequence of codon 12. G12D (c.35G>A) and G12V (c.35G>T) was the most frequent pair (8/14) of coexistent point mutations observed in NSCLCs with intratumor heterogeneity. According to the aforementioned assumption it could be the result of two consecutive point mutations in cells belonging to one clone: GGT → GAT → GTT or GGT → GTT → GAT. Such explanation is in line with the hypothesis that KRAS mutation as the one of the “driver mutations” occurs early in the NSCLC development (Alsdorf et al. 2013; Li et al. 1994). Other less frequent coexistent pairs of point mutation (e.g. G12D and G12C, G12V and G12C) could be a consequence of two additional mutations in cells of one clone or coincident KRAS mutation in the neighboring epithelial cells that subsequently developed additional cell clone co-forming NSCLC, an example of the parallel carcinogenesis.

However, our findings disagree with the results of the previous study showing homogeneous distribution of the same sequence of KRAS mutation in various regions in NSCLCs (Alsdorf et al. 2013). Moreover, this study showed the same point mutation sequence in tumor and corresponding lymph node metastases giving the impression that the phenomenon of intratumor heterogeneity of KRAS mutation in NSCLCs is very rare (Alsdorf et al. 2013). The specific study protocol of Alsdorf et al. (2013) report may explain this discrepancy. Namely, they used paraffin blocks of the primary tumor (NSCLC) and/or lymph node metastases for retrospective analysis of KRAS point mutations. Thus, the real distance between compared tumor regions could have been a subject to change and was not unequivocally established. It cannot be excluded that samples of DNA for genetic analysis were isolated from adjacent regions, hence, giving the same results in respect of KRAS point mutations. In our prospective study, tumor specimens for paraffin block preparation were always collected according to the protocol (one central and four marginal samples on a square design). However, this explanation is not applicable for the comparison of KRAS mutations between primary tumor and its lymph node metastases. Nevertheless, our results are in agreement with other studies showing: (a) multiplicity of oncogene “driver” mutations (EGFR—epidermal growth factor receptor and KRAS) in NSCLCs including coexistence of two different KRAS point mutations, one in codon 11 and the second one in codon 12 in ADC patient (Benesova et al. 2010); (b) the emergency of new oncogene mutations (KRAS in codon 13 and EGFR in exon 21) in patient with ADC who after 8 months’ response to crizotinib treatment acquired resistance to this drug with subsequent rapid disease progression (Rossing et al. 2013); and (c) different frequencies of KRAS point mutations between primary NSCLC and corresponding lymph node metastases (Schmid et al. 2009; Sun et al. 2011). Moreover, Schmid et al. (2009) described the occurrence of two different KRAS point mutations in codon 12 in one case of primary lung ADC that were also present in corresponding lymph node metastases as well as a different sequence of KRAS mutation in one case of NSCLC and its metastasis. Our results together with the aforementioned studies suggest that coexistence of two different mutated sequences in NSCLCs harboring KRAS mutation in codon 12 is not a rare and unique phenomenon.

### Implications for the usage of KRAS mutation as a cancer biomarker in EBC

We tested accordance of KRAS mutation status between EBC–DNA samples and resected NSCLC tissue in the subgroup of 19 NSCLC patients (12 with positive and 7 with negative KRAS mutation NSCLC). Only one case of discrepancy consisted in a not detectable KRAS mutation in EBC–DNA obtained from patient with KRAS mutation positive NSCLC. It is likely that DNA from distinct cancer tissue regions can contribute to cancer-derived EBC–DNA. Thus, with the proviso of inhomogeneous distribution of KRAS mutation over the cancer tissue one may expect that some samples of EBC–DNA would give false negative results, although, NSCLC as a whole, harbors this mutation. However, it should be pointed out that in four NSCLCs with inhomogeneous distribution of KRAS mutation the EBC–DNA was positive for this mutation. Moreover, the only one case of discrepancy between EBC–DNA and cancer DNA was noted in NSCLC harboring this mutation in all five studied samples. Thus inhomogeneous distribution of KRAS mutation in cancer tissue seems not to influence the result of screening of EBC–DNA for the presence of KRAS mutation. However this hypothesis should by confirmed on the larger group of patients.

Sequence analysis of KRAS point mutation in EBC–DNA revealed the same sequence as in the corresponding tumor harboring this mutation (*n* = 12). In one case of NSCLC with intratumor heterogeneity of KRAS mutation EBC–DNA sequence was the same as one of the two coexistent sequences in tumor tissue. Therefore, in such a situation comparison of mutated sequence determined in only one sample of cancer tissue with that of EBC–DNA would give discrepant result. Therefore, analysis of EBC–DNA for KRAS mutation seems promising screening method for airways carcinogenesis related to this driver mutation. Moreover, Bayesian analysis of EBC against tumor KRAS mutation status revealed high sensitivity and specificity of EBC testing. Nevertheless, these results should be accepted with caution despite significant changes in probabilities, due to the low number of cases in this sub-analysis. On the other hand, parallel comparison of NSCLC samples and blood–DNA in respect of KRAS mutation status and mutated sequences revealed lower ratio of accordance than it was observed for EBC–DNA. The cell-free DNA released from NSCLC and DNA from circulating cancer cells can contribute to high ratio of positive results of KRAS mutation in the whole blood samples. This corresponds with the results of the previous studies showing high frequency of KRAS mutation in DNA isolated from blood of patients with NSCLC bearing this mutation (Kordiak et al. 2012; Ramirez et al. 2003). DNA pool from the whole blood can also contain DNA from other sources (e.g. hematopoietic cells) and this could be responsible for discrepancies between blood and corresponding NSCLC samples in relation to KRAS mutation.

### Strengths and weaknesses of the study

Strengths of this study were the prospective design, exclusion of NSCLC patients that were treated with potentially mutagenic chemotherapy or radiotherapy, a precise protocol for tumor regions sampling for genetic analysis and random selection of patients for comparison of KRAS mutation status in cancer tissue, blood and EBC–DNA. We investigated five regions of excised NSCLC with reciprocal distance between them ranging from 0.5 to 3 cm. This ensured that samples of isolated and then analyzed DNA were not from adjacent cancerous cells.

The relatively low number of studied NSCLC patients (*n* = 51) and small subgroup of EBC and blood donors (*n* = 19) can be recognized as the weakness of the study. Intratumor KRAS mutation heterogeneity and inhomogeneous distribution over the tumor tissue were observed in colorectal cancer (Kosmidou et al. 2014) and prostate cancer (Konishi et al. 1995). We suspected that at least in some NSCLC patients, the tumor, like other aforementioned cancers, can harbor two different KRAS mutations or reveal its inhomogeneous distribution in cancerous cells. Therefore, the aim of our study was only to find the proof that this phenomenon can occur in NSCLC and to compare the KRAS mutation status between EBC–DNA samples and resected NSCLC tissue. Hence, the relatively low number of studied patients, but sufficient to show that: (a) the coexistence of two different sequences of KRAS point mutations at codon 12 is not a rare phenomenon in NSCLCs; (b) some NSCLCs reveal inhomogeneous distribution of KRAS mutation over the tumor tissue; and (c) there is a high accordance between KRAS mutation status in EBC–DNA and NSCLC tissue. Investigation of only one driver mutation (KRAS mutation) could also be recognized as a weakness of our study. However, previous studies revealed the possibility of intratumor heterogeneity of EGFR and p53 point mutation in NSCLCs (Li et al. 1994; Bai et al. 2013). Therefore, we decided to focus on analysis of KRAS point mutation at codon 12. On the other hand, another relevant candidate for such analysis would be BRAF gene because its mutations were found in subset of NSCLCs (Cardarella et al. 2013) and were detected in EBC–DNA obtained from apparently healthy subjects (Youssef et al. 2017).

## Conclusions

We found that intratumor heterogeneity of KRAS point mutation at codon 12 and inhomogeneous distribution of this mutation over the cancer tissue can occur in NSCLCs. Nevertheless, there was a high accordance between KRAS mutation status in EBC–DNA and cancer tissue in NSCLC patients. Moreover, it was higher than that between blood–DNA and cancer. These results suggest usefulness of monitoring KRAS mutation in EBC–DNA as a biomarker of NSCLC in patients harboring this mutation. However, due to low number of studied cases, it seems reasonable to confirm the value of EBC in diagnostics of KRAS mutation status of NSCLC on a larger group of patients. Moreover, the results of our study imply the relevance of planning future trials on the diagnostic impact and predictive value of oncogene mutations in exhaled DNA in heavy cigarette smokers without apparent lung cancer detectable by conventional diagnostic procedures.
